# A decade of progress: bibliometric analysis of trends and hotspots in oral microbiome research (2013-2022)

**DOI:** 10.3389/fcimb.2023.1195127

**Published:** 2023-05-12

**Authors:** Zhengrui Li, Rao Fu, Xufeng Huang, Xutao Wen, Ling Zhang

**Affiliations:** ^1^ Department of Oral and Maxillofacial-Head and Neck Oncology, Shanghai Ninth People’s Hospital, Shanghai Jiao Tong University School of Medicine, Shanghai, China; ^2^ College of Stomatology, Shanghai Jiao Tong University, Shanghai, China; ^3^ National Center for Stomatology, Shanghai, China; ^4^ National Clinical Research Center for Oral Diseases, Shanghai, China; ^5^ Shanghai Key Laboratory of Stomatology, Shanghai, China; ^6^ Shanghai Research Institute of Stomatology, Shanghai, China; ^7^ Shanghai Center of Head and Neck Oncology Clinical and Translational Science, Shanghai, China; ^8^ Faculty of Dentistry, University of Debrecen, Debrecen, Hungary

**Keywords:** oral microbiome, oral microbiota, bibliometric analysis, oral health, oral diseases

## Abstract

**Background:**

Over the past decade, a plethora of studies have delved into the oral microbiome. Our objective was to evaluate the trends in oral microbiome research employing a quantitative approach.

**Materials and methods:**

We extracted clinical studies on the oral microbiome published between 2013 and 2022 from the Web of Science database, yielding 3024 articles. The assembled literature was visually scrutinized using VOSviewer 1.6.18, Citespace 6.1.6, Pajek, Scimago Graphica, and other specialized software to assess authors, institutions, countries, journals, co-cited literature, keywords, genes, and diseases.

**Results:**

Our analysis identified a total of 3024 articles. The volume and rate of annual publications steadily increased, with research interest in the oral microbiome progressively intensifying. The United States, China, and the UK contributed the highest number of publications. Growth rates of publications varied among countries over time. The Forsyth Institute emerged as the most collaborative institution, boasting the highest number of relevant papers (135) and securing the top rank, followed by Sichuan University and Harvard University. Paster Bruce J, Zhou Xuedong, and He Xuesong were pioneers in the field of oral microbiome research. This analysis demonstrates that the homeostatic balance of the oral microbiome, advanced microbial sequencing technology, connections with gut microbiota, and tumorigenesis, including oral cancer, have become emerging topics in the oral microbiome field.

**Conclusions:**

This study delineated a comprehensive landscape of hotspots and frontiers in oral microbiome research, thus facilitating the identification of interdisciplinary advancements. We sincerely hope that our bibliometric analysis will enable researchers to leverage the oral microbiome to ultimately improve human oral health.

## 1 Introduction

The term “oral microbiome” encompasses the entire oral cavity, including microbes, genomes, and the surrounding microenvironment, collectively known as the microbiota (Li et al., 2022). The human oral cavity hosts a diverse array of microorganisms, including bacteria, fungi, viruses, archaea, and protozoa (Zhang et al., 2018). The oral microbiome is the second most abundant microbiome after the gastrointestinal tract, with a direct impact on human health, from host metabolism to immune responses (Ritchie et al., 2016). The human microbiome’s crucial role in maintaining homeostasis and limiting the development of diseases and cancer (Helmink et al., 2019) has sparked significant interest in the oral microbiome.

Advances in metagenomics and next-generation sequencing techniques, such as 16S rRNA sequencing, have enabled in-depth exploration of the oral microbiome. Subsequent studies have investigated the relationship between the oral microbiome and oral/systemic diseases. Research has demonstrated that the oral microbiome is not only associated with oral diseases such as dental caries and periodontal disease (Colombo and Tanner, 2019) but is also closely related to systemic diseases such as diabetes, cardiovascular disease, rheumatoid arthritis, premature delivery, respiratory disease, colorectal cancer, inflammatory bowel disease, and Alzheimer’s disease (He et al., 2015a; Flemer et al., 2018; Xu et al., 2018). The role of the oral microbiome in the pathogenesis and progression of diseases cannot be overlooked and has attracted widespread attention and research. Numerous studies on the oral microbiome aim to ultimately improve human oral health.

As scientific publications have proliferated over time, the rapid succession of oral microbiome discoveries in the past decade has had a widespread impact and contributed to a large number of highly cited articles. This study presents a bibliometric and visualized analysis of trends in oral microbiome research. Bibliometric analysis is a quantitative approach that identifies current research characteristics and trends in a specific field by searching a literature database (Farias da Cruz et al., 2022), typically involving the Web of Science database (Shen et al., 2019). However, quantitative studies providing a comprehensive analysis of developmental trajectories in this field are scarce. The aim of this bibliometric analysis is to review the oral microbiome and elucidate research trends and hotspots in the oral microbiome field.

## 2 Materials and methods

### 2.1 Data collection

In our study, we conducted a systematic analysis of publications from January 1st, 2013, to December 31st, 2022, to delineate the evolving landscape of oral microbiome research. Data for this bibliometric analysis were obtained from the Web of Science Core Collection (WoSCC) database. We performed a search using a combination of the following keywords and terms: (“oral microbiome” OR “oral microbiota” OR “oral microbiology” OR “oral microbe” OR “oral microorganism”). The inclusion criteria encompassed papers and reviews related to the search while excluding letters, newsletters, book reviews, and other non-research materials. A total of 3725 articles were initially retrieved.

**Figure 1:**
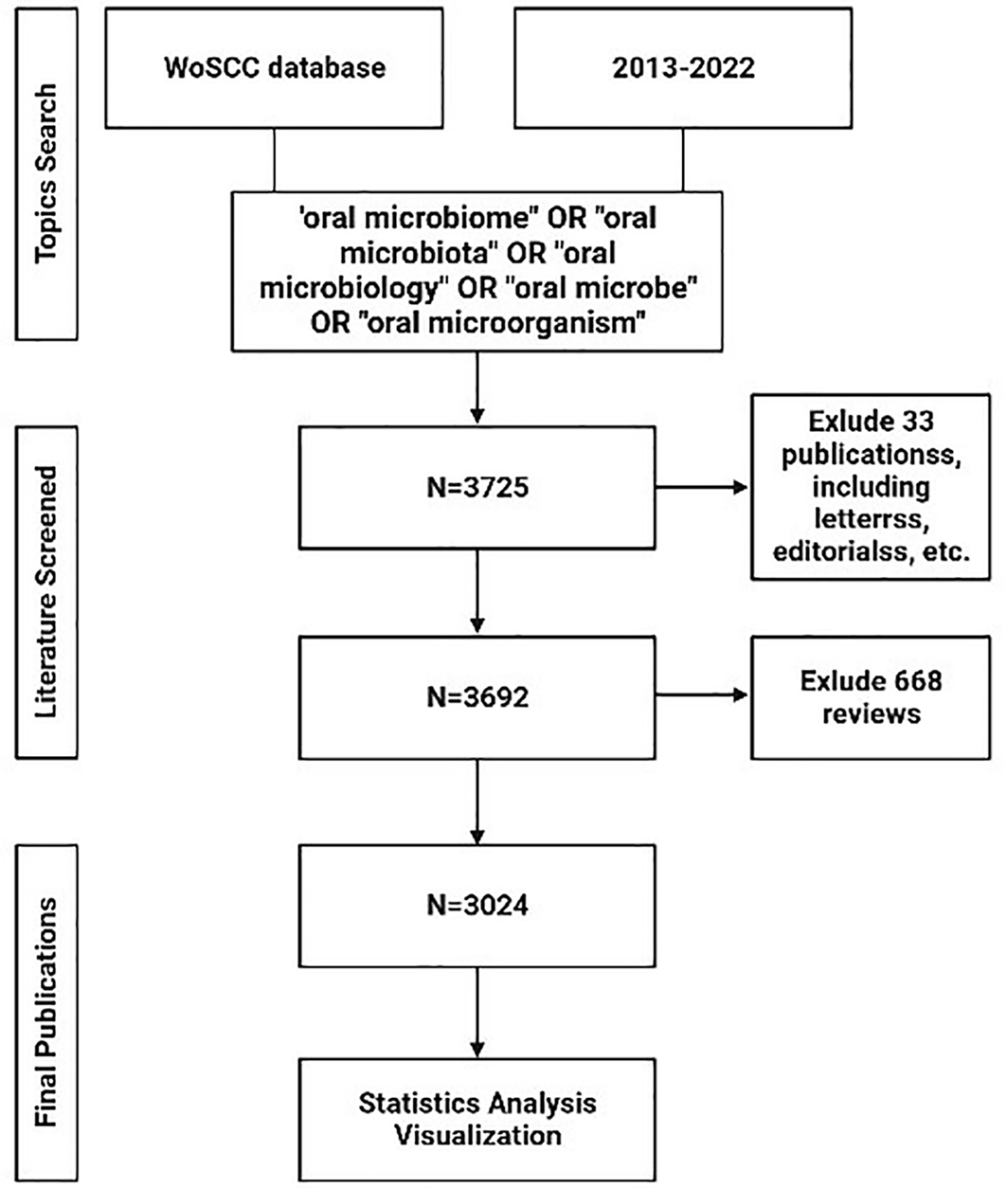
Flowchart of literature collection and selection.

After excluding 23 conference papers, 1 data paper, 9 books and 668 reviews, the remaining dataset consisted of 3024 articles. This dataset was used for visual analysis of authors, institutions, countries, journals, co-cited references, and keywords to elucidate the trends and patterns in the field of oral microbiome research (**Figure 1**).

### 2.2 Statistics analysis and visualization

The retrieved literature was visually analyzed using VOSviewer 1.6.18 (van Eck and Waltman, 2010), Citespace 6.1.6 (Shen et al., 2019), Pajek, Scimago Graphica, and other software tools to assess authors, institutions, countries, journals, co-cited literature, keywords, genes, and diseases. The information on genes and diseases was obtained from New Big Data Analysis Platform (https://www.citexs.com/Advanced). Relevant visual maps were created to examine the current research status, hotspots, and trends in the field of oral microbiome research. This comprehensive analysis aimed to provide insights into the evolving landscape of this rapidly growing field.

## 3 Manuscript formatting results

### 3.1 Annual issuance volume and its trend

**Figure 2:**
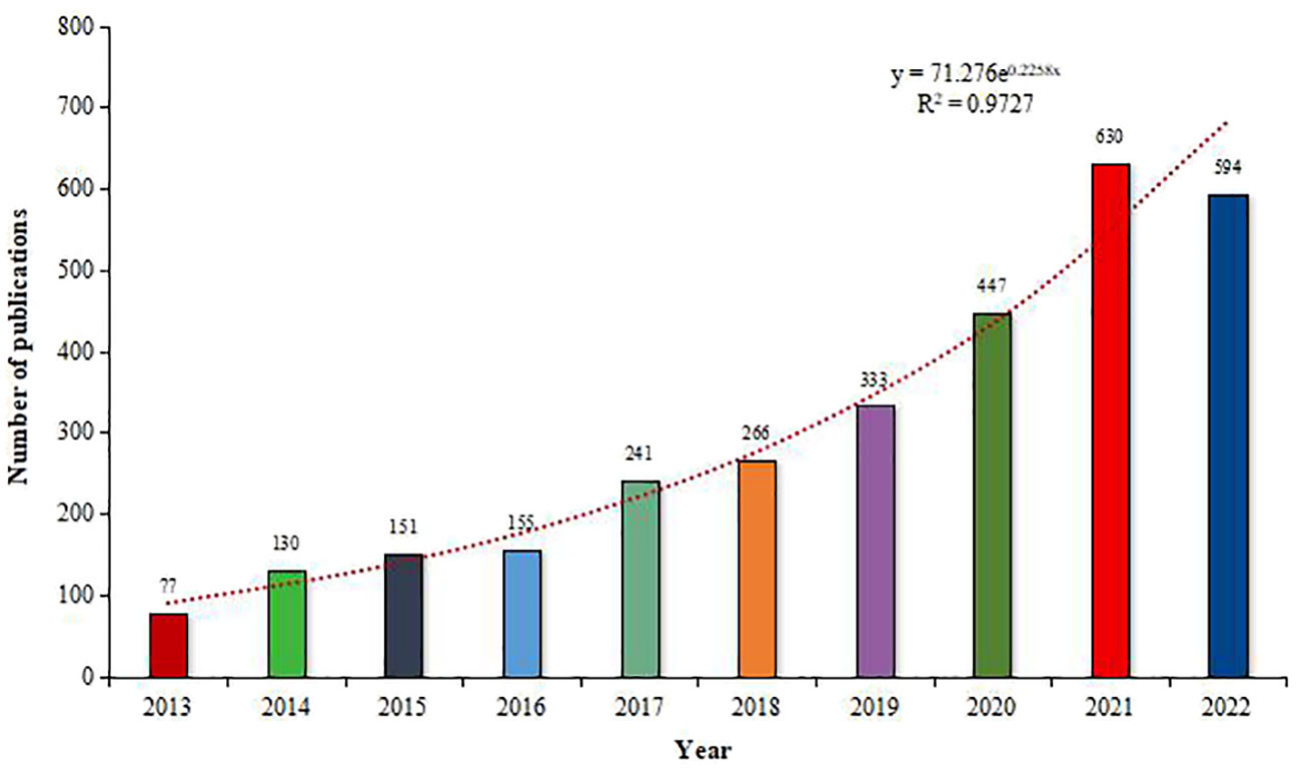
Publications on oral microbiome-related research (increasing trend) between 2013 and 2022.

From 2013 to 2022, a total of 3024 relevant articles on the oral microbiome were included, with an average annual publication count of 302.4 and an average growth rate of 31.7%. Over the past decade, the number of papers in this field has generally demonstrated a steady upward trend, indicating that the research interest in the oral microbiome has been increasing year by year and holds significant research value. However, in 2016, the growth rate was not substantial, and the publication count remained almost the same as the previous year. An exponential function, y = 71.276e^0.2258x^ (R^2 = ^0.9727, where x represents the year, and y represents the annual publication volume), was used to further model the annual publication trend, and the curve fitting was satisfactory (**Figure 2**). Nonetheless, the number of publications in 2022 did not achieve a further breakthrough, which could be attributed to factors such as the COVID-19 pandemic, publication lag, and other reasons.

### 3.2 Research region and its relationship

**Figure 3:**
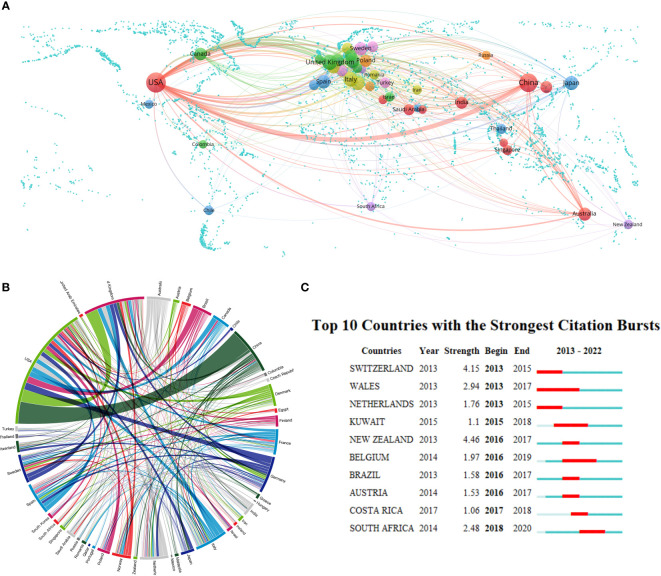
Country/region collaboration in the field of oral microbiome research. **(A)** Cooperation geo-heat map and its co-occurrence network. **(B)** Country/region partnership chord maps. **(C)** The number of documents issued on oral microbiome research highlights Top10 countries (red areas indicate the time period when documents have surged).

A total of 99 countries/regions have conducted oral microbiome-related studies. Setting the minimum number of published documents per country at 10, we obtained a national collaboration diagram for oral microbiome research (**Figures 3A, B**). We performed a visual analysis of the publishing regions. In the geographic map, each sphere represents a country, and the color of each sphere represents the clustering relationship between the study areas, which are clustered according to the intensity of mutual collaboration, and are divided into seven clusters. The width of the lines connecting the spheres indicates the intensity of collaboration between countries; the size of the spheres is directly proportional to the number of published documents per country. Each peripheral curve segment in the chord diagram represents a country, with longer peripheral curve segments indicating more publications from that country, and the thickness of the connections being directly proportional to the intensity of cooperation between countries. The United States has the highest number of published documents, with 1071 publications, accounting for 33.43% of the total, and demonstrates the strongest willingness to cooperate with other countries; followed by China and the United Kingdom, accounting for 19.85% and 7.43%, respectively (**Supplementary Table 1**). Interestingly, the United States and China ranked first in the total number of publications, but did not rank high in centrality. In contrast, Italy and Germany have established cooperative relationships (high centrality) with many regions, despite contributing only a few articles. This may be related to the differing academic atmospheres in Europe and Asia, with European countries favoring multi-country, multi-institutional joint research.

We further analyzed the number of oral microbiome research publications from 2013 to 2022 (**Figure 3C**). Countries such as the United States and China, with a higher number of publications, have not experienced a sudden increase in publication count, which is attributable to the consistent scientific research and output capabilities of researchers in these countries. From 2013 to 2015, Switzerland experienced a surge in publications. Wales had the longest period of increased publication count, from 2013 to 2017. In the past five years, countries exhibiting a surge in publication count include Costa Rica and South Africa, indicating their focus on oral microbiome research during this period. This also suggests that dentistry and related research are no longer exclusive to developed countries in Europe and the United States, as African countries have increasingly begun to pay attention to oral microbiome-related research. However, research in this field remains absent in some regions and needs further development. This demonstrates that, as experimental technology and scientific research methodologies continue to evolve, more countries are recognizing the importance of oral microbiome research.

### 3.3 Research institutions

**Figure 4:**
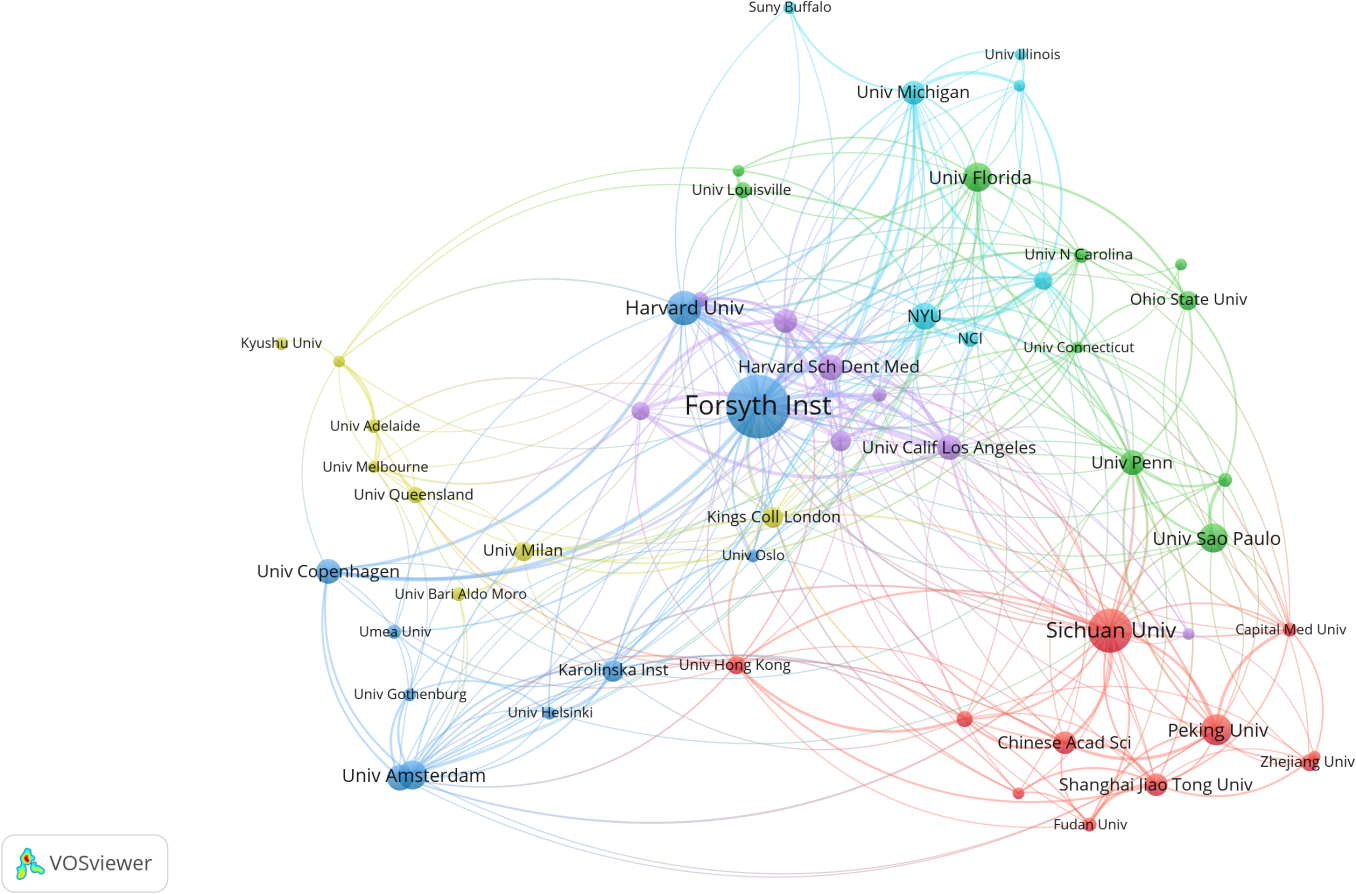
Institutional collaboration in the field of oral microbiome. Collaborative clustering networks of relevant research institutions.

Over the past decade, a total of 3,435 institutions have conducted oral microbiome-related studies; however, the majority have not pursued continuous research. By setting a minimum publication threshold of 38 documents per institution, we obtained the top 20 research institutions’ cooperation relationship maps and clustering maps (**Figure 4**). We brought together institutions with similar research collaboration patterns and divided them into six clusters. The size of the circles is directly proportional to the number of publications. Different colors of circles represent different clusters, and the shade of the color of the line connecting the circles indicates the strength of cooperation between institutions. We found that the Forsyth Institute (135), Sichuan University (89), and Harvard University (67) have made significant contributions to oral microbiome research. Among the top ten institutions, five are American, and two are Chinese, occupying the top five positions (**Supplementary Table 2**).

Regarding inter-institutional collaboration, we found that the Forsyth Institute has a leading centrality and demonstrates the strongest willingness to cooperate with other institutions. This is evidenced by the close connections between the Forsyth Institute of Stomatology, affiliated with Harvard Medical School, and nearly all high-volume institutions. Although Sichuan University has published a large number of documents, it has not engaged in intensive inter-institutional collaboration. This may be related to China’s cooperation and exchange habits, as it tends to collaborate more with local institutions.

### 3.4 Research authors

**Figure 5:**
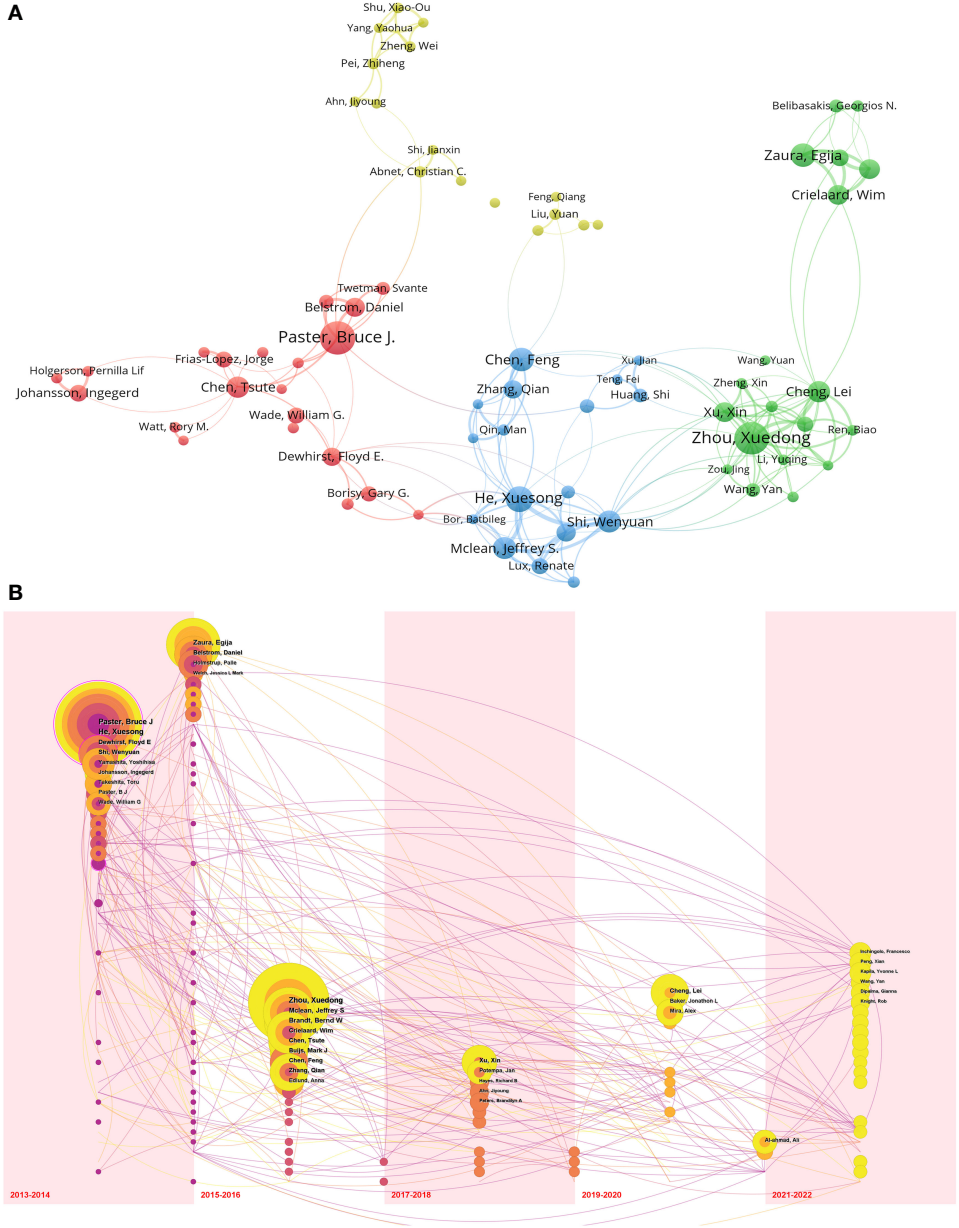
Cooperation of authors in the field of oral microbiome. **(A)** Cooperation network between authors. **(B)** Temporal overlay of the author ‘s cooperative network.

Through an analysis of authorship data, we identified a total of 16,676 authors who contributed to publications related to the oral microbiome. Each author published a minimum of eight documents. The size of the circles in the visual representation is directly proportional to the number of documents issued by each author, while the colors denote different clusters. The thickness of the lines between circles indicates the strength of collaboration between authors. A cooperation diagram for oral microbiome-related investigators was generated (**Figure 5A**).

Most of the top 10 scholars in oral microbiology are from the United States (**Supplementary Table 3**). The top three authors in terms of publication volume were Paster Bruce J (The Forsyth Institute, 32 publications), Zhou Xuedong (Sichuan University, 31 publications), and He Xuesong (University of California until 2017/The Forsyth Institute from 2017 onwards, 24 publications). Based on the strength of connections among the top 74 high-output authors, their collaborations were divided into four clusters, led by The Forsyth Institute (represented by Paster Bruce J) and Sichuan University (represented by Zhou Xuedong). Within each author group, close collaboration patterns emerged. For example, Zhou Xuedong and Cheng Lei from Sichuan University have a strong cooperative relationship. Paster Bruce J from The Forsyth Institute closely collaborates with Belstrom Daniel, while He Xuesong has strong collaborations with Shi Wenyuan and Mclean Jeffrey S. These patterns may suggest that advanced teams in the field of oral microbiome research have well-defined internal divisions.

We also examined nodes within the same time zone. Each sphere in the visual representation denotes an author, with size directly proportional to their publication volume, while the time zone corresponds to the year of their first publication. Purple indicates that an author published relatively early, yellow suggests recent publication, and superimposed colors indicate publication in the corresponding year. A higher number of superimposed colors form an annual wheel, representing the prolific and continuous publication of an author’s work. Connections between nodes within the time zone indicate collaborative relationships among authors (**Figure 5B**). A circle marked with a rosy edge line represents an author with a high number of citations. The top three authors, Paster Bruce J, Zhou Xuedong, and He Xuesong, are pioneers in the field of oral microbiome research and have achieved numerous significant research results in the early stages, thus inspiring subsequent research by other scholars. Furthermore, Zhou Xuedong and He Xuesong continue to conduct research and publish findings.

### 3.5 Journal and cited journal

**Figure 6:**
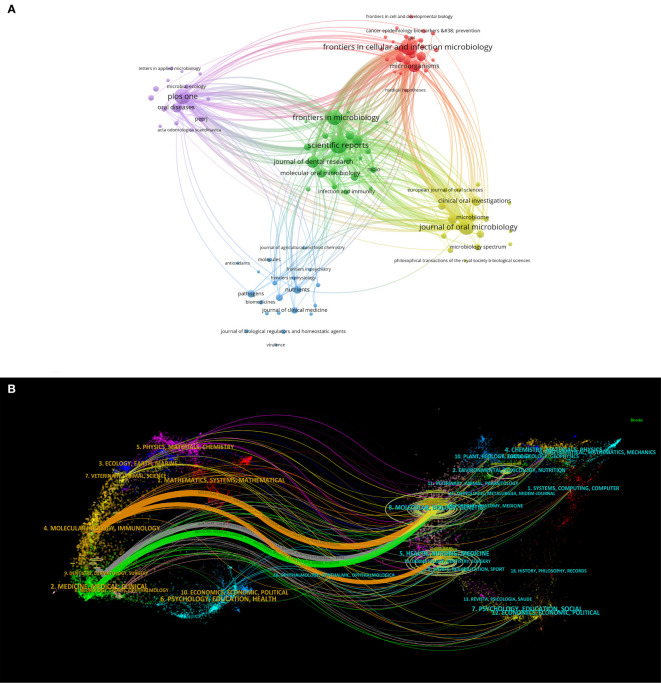
Journal analysis in the field of oral microbiome. **(A)** Journal clustering visualization map. **(B)** Biplot overlay of journal.

A visualized analysis of journal publications revealed that 792 journals featured articles on oral microbiome-related research. Setting the minimum number of documents issued by each journal at 5, we obtained a cluster diagram of documents issued by these journals. The circle size is positively correlated with the number of journal documents issued (**Figure 6A**), and the color represents different clusters, which are divided into five clusters. Among the top ten journals, Frontiers in Cellular and Infection Microbiology (129) has the highest number of issued documents, followed by others such as Scientific Reports (127) and Journal of Oral Microbiology (112). The top ten journals include five microbial journals and four dental medical journals. Seven are JCR Q1 journals, and five have impact factors > 5, including Frontiers in Cellular and Infection Microbiology (6.073), Journal of Oral Microbiology (5.833), Frontiers in Microbiology (6.064), Journal of Dental Research (8.924), and Periodontology 2000 (12.239). This indicates that oral microbiome-related articles are of high quality and possess significant academic value.

Additionally, we performed a biplot superposition of the journals (**Figure 6B**). The figure is divided into two parts: the left side represents the citing journals and the right side represents the cited journals. The results show the position of oral microbiome theme research relative to major research disciplines. Each point on the graph represents a journal, and the curves between the left and right parts of the map indicate citation connections. The trajectories of these connections provide insight into interdisciplinary relationships in the field and reveal the context of citations. Our findings suggest that oral microbiome research has consistently been closely related to major research disciplines such as molecular biology, immunology, clinical medicine, dentistry, and oral surgery, and has remained integrated with mainstream research.

**Figure 7:**
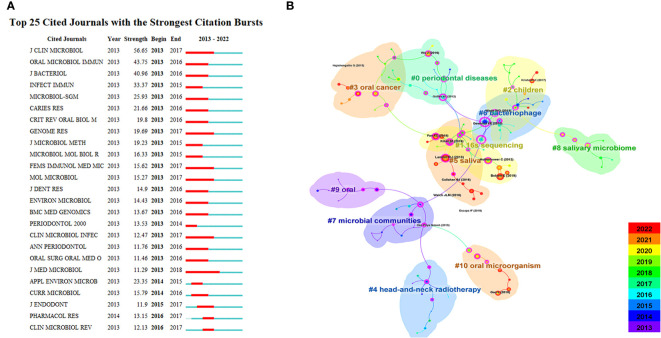
Journal analysis in the field of oral microbiome. **(A)**. Top25 cited journals on oral microbiome. **(B)**. Literatures on oral microbiome are co-cited in clusters.

The indexed journals of oral microbiome between 2013 and 2022 (**Figure 7A**) are further analyzed, along with journal citation figures on oral microorganisms. Journal citation figures refer to the surge in the number of citations of a particular journal during a certain period, with the red area in the figure indicating the time period. During this time, multiple journals, such as the Journal of Clinical Microbiology, experienced a surge in citations from 2013 to 2017, demonstrating that much of the research literature on the oral microbiome originated from these sources.

CiteSpace was used to analyze the citation of oral microbiome literature over the past decade and the top 10 cited article (**Supplementary Table 4**). The keyword cluster analysis atlas included 125 nodes and 134 lines (**Figure 7B**). The cluster module value Q = 0.8277 and the average silhouette value S = 0.9548. CiteSpace provides the module value (Modularity, Q value) and the average silhouette value (Mean Silhouette, S value) based on network structure and clustering clarity. Consequently, from the upper left region of **Figure 6**, Q value = 0.8277 and S value = 0.9546, it can be concluded that the sample keyword clustering structure is significant and the clustering is convincing (generally, when Q > 0.3, it indicates that the clustering structure is significant, and when S > 0.5, it indicates that the clustering is reasonable). These citations were clustered into 11 categories, including 16S sequencing, children, oral cancer, and head and neck radiotherapy.

Over time and with research progress, researchers have gradually shifted their focus to the individual role of microbial communities, to the oral microbiome at different sites (such as the parotid gland and tongue), and to disease treatment. Subsequently, research has concentrated on the relationship between the oral microbiome and oral diseases as well as oral tumors. This trend demonstrates that the research focus has transitioned from treatment to prevention, exploring the key role of the oral microbiome in disease development, and identifying microbial markers and targets for various diseases.

### 3.6 Keywords

**Figure 8:**
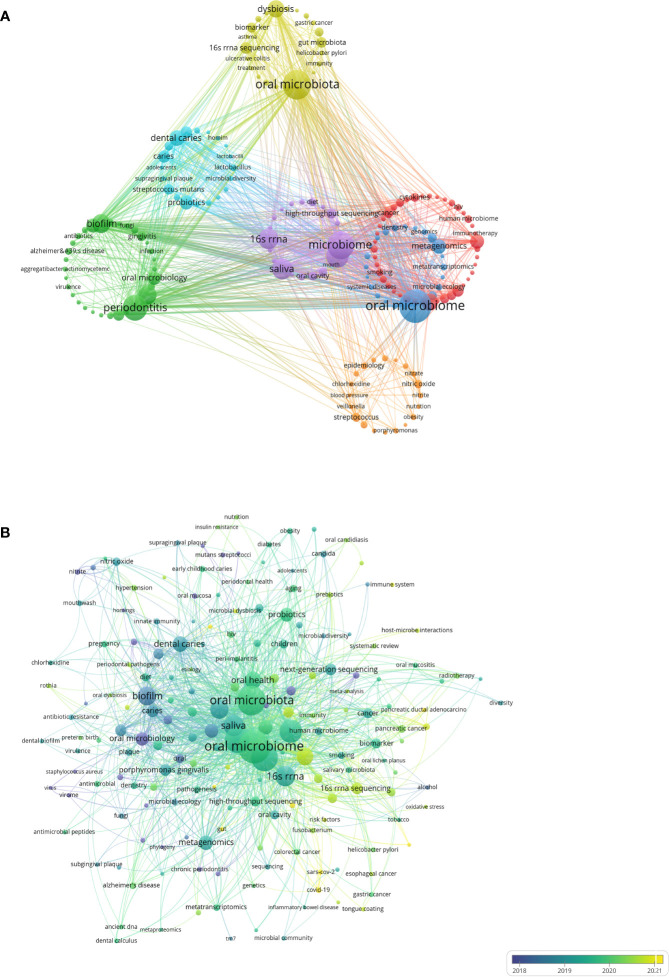
Keywords analysis in the field of oral microbiome. **(A)** Keywords clustering visualization. **(B)** Keywords intensity visualization timing overlay.

Keyword analysis reflects the current state of research topics in the oral microbiome field, including hotspots and future directions. Through co-occurrence cluster analysis of keywords, the minimum occurrence frequency for each keyword was set to 8 times. After cleaning 5,234 keywords, removing meaningless words, combining synonyms, and screening 182 keywords, a visual map was created (**Figure 8A**). Circles and labels form nodes, with circle size positively correlated with keyword frequency and circle line thickness positively correlated with the strength of relationships between keywords. Nodes of different colors form different clusters, with each color representing a distinct research direction, divided into 7 clusters.

Yellow clusters represent the direction of oral microbiota research. While overall oral microbiota research remains the main form of relevant research, the distal connection between microbiota is gradually gaining researchers’ attention. Green clusters are focused on periodontitis research (including periodontitis, periodontal disease, biofilm bacteria, oral microbiology, and Porphyromonas gingivalis), while light blue clusters are centered on dental caries research (including dental caries, probiotics, caries, dental plaque, and Streptococcus mutans). Oral diseases such as periodontitis, caries, and gingivitis are risk factors for various systemic diseases, and their relationship with the oral microbiome has long been a research focus.

Blue clusters represent the direction of oral microbiome research, with biological function and metabolic pathways as important research directions, helping to understand the involvement of the oral microbiome in physiological and pathological body processes (including oral microbiome, metagenomics, and metabolomics). Red clusters are focused on oral health research (including oral health, inflammation, next-generation sequencing, and cancer), while purple clusters represent microbiome research directions (including microbiome, microbiota, saliva, and 16S rRNA). The keywords “oral microbiome,” “oral microbiota,” and “microbiome” ranked in the top three, with 491, 392, and 350 occurrences, respectively.

The”Oral microbiota” represents the collection of microorganisms present in a specific environment (oral cavity). The emphasis is on the importance of microorganisms associated with human health and disease. The study of microbial composition is mainly done by molecular methods, based on the analysis of 16S, 18S rRNA or other marker genes or genomic regions, amplification of biological samples, sequencing and finally classification into different phyla based on sequences.

The “Microbiome” refers to the entire genome (genes) of microorganisms (bacteria, archaea, lower or higher eukaryotes and viruses), including their surroundings. This definition is based on ‘biome (biotope)’ and includes all biological and microbial elements in the environment. The “Microbiome” is characterized by a combination of macrogenomics, metabolomics, macrotranscriptomics, and macroproteomics, and a collection of clinical/environmental data. The microbiome in this context is more like the microbiome of the organism as a whole. The “Oral microbiome” is defined as the entire genome (genes) of microorganisms (bacteria, archaea, lower or higher eukaryotes, and viruses), and their surroundings, within the purely oral environment. The “Oral microbiology” is the science that studies microorganisms in the oral cavity and their role in oral health and disease. The field is concerned with microbial interactions, relationships with hosts, and methods for the prevention and treatment of oral diseases.

The mean year of emergence for different keywords is visualized with colors (**Figure 8B**). Blue indicates earlier keywords, and yellow indicates recent keywords. Microbial studies of oral diseases emerged early, followed by oral microbiota and oral microbiome research that focused on oral microorganisms as overall study subjects. Over time, researchers have gradually shifted away from single diseases and single microorganisms, but with the help of cutting-edge microbiomics (including microorganisms, genomics, and even the environment and groups), indicating that the overall research direction of the oral microbiome has become the most prominent topic. Association studies of oral microorganisms with tumors and other systems represent recently emerging keywords and have the potential to become a new research direction.

**Figure 9:**
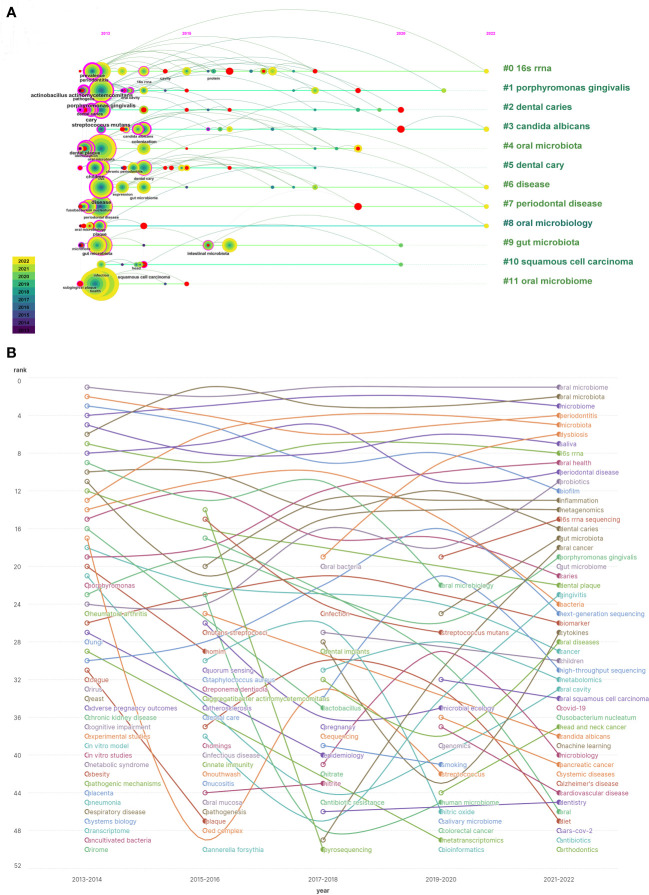
Keyword timing analysis in the field of oral microbiome. **(A)** Co-occurrence of temporal trends in keywords. **(B)** Keyword heat trend graph.

Timeline analysis after clustering keywords associated with the oral microbiome reveals that purple represents keywords that appear relatively early, yellow represents recently emerged keywords, and superimposed colors represent the appearance of keywords in corresponding years. Red nodes are key nodes, and keywords of the same cluster are placed on the same horizontal line. The closer the keywords appear to the top of the view, the further right they are. This figure allows us to determine the number of keywords in each cluster, with more keywords indicating greater importance in the clustering field. The time span of keywords in each class can also be observed. As shown, the keywords are clustered into 12 clusters (**Figure 9A**), including 16S rRNA (#0), Porphyromonas gingivalis, dental caries, Candida albicans (#3), oral microbiota, dental caries, disease (#6), periodontal disease (#7), oral microbiology (#8), gut microbiota, squamous cell carcinoma, and oral microbiome. Clusters #0, #3, #6, #7, and #8 continue to develop in the field of oral microbiome research, but new research hotspots should not be underestimated.

By examining keywords from articles published between 2013 and 2022, the frequency and ranking of keywords were counted every two years, yielding a heat trend chart of keywords over the past decade (**Figure 9B**). The ranking trend of curve fluctuations is also shown. Oral microbiome, oral microbiota, and microbiome have consistently been research priorities over the past decade. Keywords such as dysbiosis, oral health, 16S rRNA sequencing, gut microbiota, and oral cancer are on the rise. Open circles indicate the time periods when a keyword first appeared in nearly a decade of research, while filled circles represent the time periods when a keyword ended.

This analysis demonstrates that the homeostasis balance of the oral microbiome, macroscopic effects on oral health, high-throughput microbial sequencing technology, close connections with gut microbiota, and tumorigenesis, including oral cancer, have become new topics in the oral microbiome field. These emerging areas will help guide future research efforts.

### 3.7 Key genes and diseases

**Figure 10:**
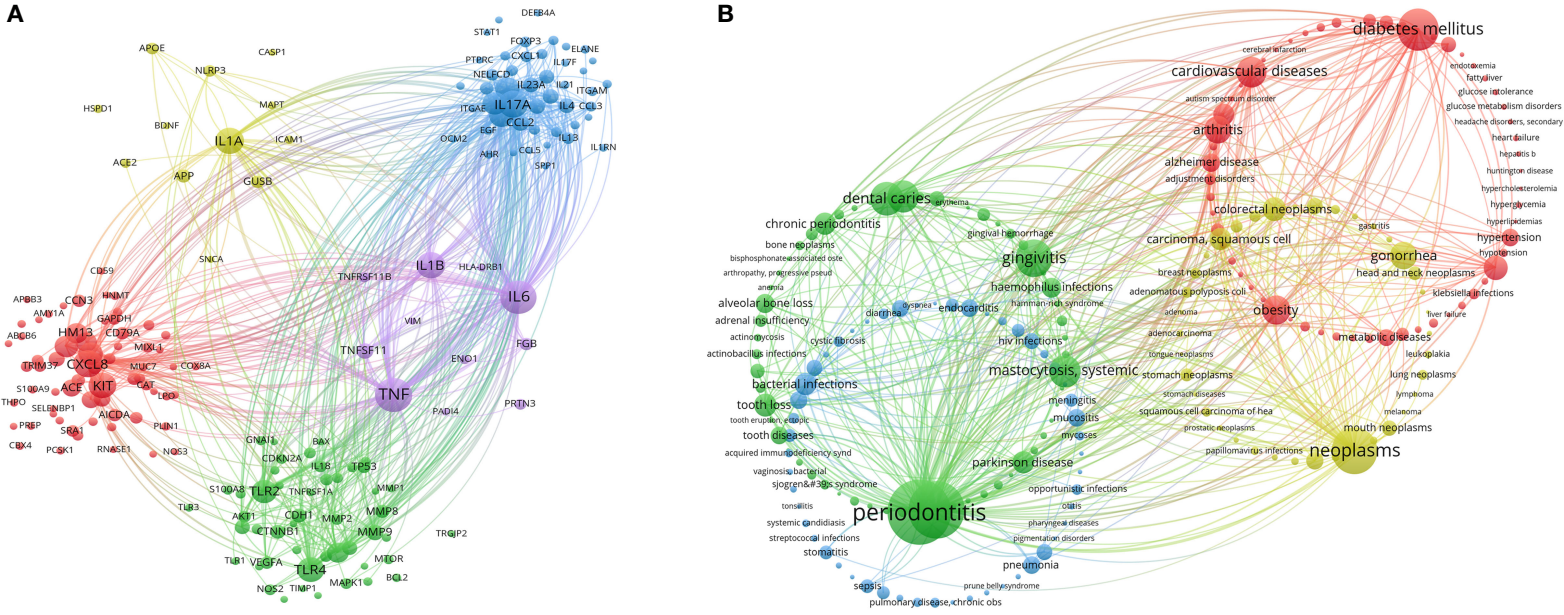
Critical genes and diseases analysis in the field of oral microbiome. **(A)** VOSviewer Critical Gene Clustering Visualization. **(B)** VOSviewer Critical Disease Clustering Visualization.

Critical genes and diseases analysis helps researchers new to the field quickly understand important gene targets and diseases related to oral microbiome research. Through co-occurrence cluster analysis of genes related to oral microbiome research, a minimum number of occurrences for each gene was set to 10 (relevant genes meeting the above conditions were included in the figure) to form a visual map (**Figure 10A**). Circles and labels form a node, with the size of circles positively correlated with the frequency of gene occurrence, and the thickness of circle lines positively correlated with the strength of the relationship between genes. Nodes of different colors form different clusters, with different colors representing gene clusters in different fields (specific cluster names need to be summarized by themselves). Red clusters represent the chemokine field, with the highest heat for CXCL8; yellow and blue clusters represent the immune-related interleukin field, with the highest heat for IL1A, IL17A, and KIT; green clusters represent the inflammation-related Toll-like receptors (TLRs) field, with the highest heat for TLR4 and TLR2; purple clusters represent the tumor necrosis factor (TNF) field, with the highest heat for TNF, IL6, and IL1B. At present, research on oral microbiome and their gene targets has shifted focus from the traditional direction of inflammation to fields such as immunity and cancer.

Through co-occurrence cluster analysis of related diseases in the field of oral microbiome, a minimum number of 25 occurrences of each disease was set to form a visual map (**Figure 10B**). Circles and labels form a node, with the size of circles positively correlated with the frequency of disease occurrence, and the thickness of circle lines positively correlated with the strength of the relationship between diseases. Nodes of different colors form different clusters, with different colors representing disease clusters in various fields, divided into a total of four clusters. The green cluster is in the field of oral non-neoplastic diseases (especially inflammatory diseases), with the highest heat for periodontitis, periodontal diseases, gingivitis, and dental caries; the blue cluster is in the field of infectious diseases, with the highest heat for bacterial infections and pneumonia; the red cluster is in the field of systemic diseases, with the highest heat for diabetes mellitus and cardiovascular diseases; the yellow cluster is in the field of tumors, with the highest heat for neoplasms, gonorrhea, colorectal neoplasms, and carcinoma, squamous cell. Researchers in the oral microbiome field have shifted their focus from traditional oral diseases to exploring associations between oral microbiome and systemic diseases, especially infectious diseases and tumors.

## 4 Discussion

Our analysis of the oral microbiome research landscape from 2013 to 2022 reveals a consistent upward trend in annual publication volume, totaling 3024 relevant articles and averaging 302.4 publications per year. This growth, reflected by an average rate of 31.7%, highlights the burgeoning research interest in the oral microbiome and its substantial value in scientific investigations. However, an exception occurred in 2016, when the number of published articles slightly decreased instead of maintaining the original growth momentum. The steady increase in publications underscores the oral microbiome’s pivotal role in human health and the expanding opportunities for interdisciplinary collaboration and innovation. The United States and China, which published the most papers, have made significant contributions to the study of oral microorganisms. However, the number of references and H-index of literature cited by American institutions and scholars are notably higher than those of Chinese scholars, indicating that the United States has maintained a high level of research output, while China is a rising power. Despite China’s rapid growth and dominance in the field, there is still a need for greater regional cooperation and academic influence.

Our analysis of institutional contributions reveals a diverse landscape of research institutions, with 3,435 institutions conducting oral microbiome-related studies over the past decade. However, a majority of these institutions have not maintained consistent research efforts in this field, suggesting that future efforts should focus on fostering sustainable research collaborations and promoting a long-term research agenda. We identified the top 20 research institutions, which were mapped and clustered according to their cooperation relationships. Institutions such as the Forsyth Institute, Sichuan University, and Harvard University emerged as significant contributors to oral microbiome research. The dominance of American institutions, with five out of the top ten, emphasizes the prominent role of the United States in this research area. In terms of inter-institutional collaboration, the Forsyth Institute displayed a leading centrality and the strongest willingness to cooperate with other institutions. The institution’s affiliations with Harvard Medical School and various high-volume institutions support the notion that strong connections between research institutions can facilitate collaboration and drive research advancements. Conversely, while Sichuan University exhibited a high publication output, it lacked intensive inter-institutional collaboration. This observation may be attributed to China’s collaboration patterns, which tend to favor local institutions rather than engaging in broader international cooperation. Future efforts should explore strategies to foster more robust collaborations between Chinese institutions and their international counterparts to facilitate a more global exchange of ideas, resources, and expertise. In this way, understanding the landscape of research institutions and their collaboration patterns is crucial for advancing the oral microbiome field. Encouraging sustainable research efforts, fostering global collaborations, and leveraging the strengths of leading institutions will be essential in driving innovation and ultimately improving human oral health.

Our analysis of authorship data for oral microbiome publications identified 16,676 authors, with the top contributors primarily hailing from the United States. The visual representation of author collaborations revealed four main clusters of authors, led by the Forsyth Institute and Sichuan University. The strong collaboration patterns within each author group suggest that well-established research teams in the oral microbiome field have clearly defined internal divisions, which may contribute to the effectiveness and impact of their research. The examination of nodes within the same time zone revealed that the top three authors, Paster Bruce J, Zhou Xuedong, and He Xuesong, were pioneers in the field of oral microbiome research. Bruce Paster J is the most published and influential scholar in the field (Aas et al., 2005; Dewhirst et al., 2010). Zhou Xuedong is mainly engaged in oral microbiology and microecology, etiology and prevention of dental pulp diseases (Zheng et al., 2021; Zhou et al., 2021; Peng et al., 2022). He Xuesong focuses on oral microbial ecology, Oral microorganisms are associated with local and systemic diseases, General microbial and system health, Probiotics and oral health (He et al., 2015b; Baker et al., 2017; Baker et al., 2019; Chipashvili et al., 2021). They has been leading the development of oral microbiology for more than 20 years. Their continuous publication of findings and significant research achievements in the early stages of the field have inspired subsequent research efforts by other scholars. This highlights the importance of the ongoing contributions of leading researchers in maintaining the momentum of the field. Analysis of journal publications showed that 792 journals featured articles on oral microbiome research, with the majority of top-issuing journals having high impact factors. This observation underscores the high quality and academic value of oral microbiome research. Furthermore, the biplot superposition of journals suggested that oral microbiome research has consistently been related to major research disciplines, emphasizing the interdisciplinary nature of the field and its integration with mainstream research. Our analysis of indexed journals of oral microbiome research and top-cited articles revealed a shift in research focus over time. Researchers have moved from investigating the individual role of microbial communities to exploring the oral microbiome’s role in disease development (Sedghi et al., 2021) and identifying microbial markers and targets for various diseases (Zhang et al., 2019; Kleinstein et al., 2020). This trend indicates a transition from treatment-focused research to prevention-focused research (Gaffen and Moutsopoulos, 2020; Sureda et al., 2020; Gebrayel et al., 2022), which may lead to more targeted and effective interventions in the future. The strong collaborative patterns among researchers, interdisciplinary connections, and the shift in research focus from treatment to prevention collectively suggest that the field of oral microbiome research is poised for significant advancements in the coming years. The bibliometric analysis results presented above provide a comprehensive overview of the oral microbiome research landscape, highlighting major research directions, trends, and potential future areas of exploration. Based on these findings, we can discuss several important aspects and implications for the field of oral microbiome research.

First, our analysis shows a significant shift in research focus from individual microorganisms and single diseases to a more holistic approach, examining the overall oral microbiome and its complex interactions with various diseases and health conditions (Wade, 2013; Gao et al., 2018). This transition is reflected in the emergence of cutting-edge microbiomics research, which integrates various fields such as genomics (Balachandran et al., 2020), metagenomics (Pasolli et al., 2019), and metabolomics (Takahashi, 2015). This multidisciplinary approach is crucial for understanding the intricate interplay between the oral microbiome and its host, as well as the implications for human health.

Second, our results highlight the increasing importance of the oral microbiome in the context of systemic diseases and conditions (Graves et al., 2019), such as diabetes mellitus, cardiovascular diseases, infectious diseases, and various types of cancer. This expanding body of research suggests that the oral microbiome may play a crucial role in the development and progression of these conditions, potentially serving as a target for therapeutic interventions and preventative strategies. The relationship between the oral microbiome and diabetes development has been well-established (Long et al., 2017; Kunath et al., 2022; Qin et al., 2022), particularly the connection between periodontal disease caused by oral microbial dysbiosis and diabetes, which are closely related and mutually influential. Diabetes leads to alterations in oral bacterial composition, and the oral microbiota of diabetic mice demonstrates increased pathogenicity when transferred to germ-free mice (Hosomi et al., 2022). Moreover, treatment with IL-17 antibodies reduces the pathogenicity of the oral microbiota in diabetic mice (Xiao et al., 2017). The oral microbiota from IL-17-treated donors results in reduced neutrophil recruitment, diminished IL-6 and RANKL levels upon transfer to recipient germ-free mice, and decreased bone resorption. Enhanced IL-17 in diabetes alters the oral microbiome, increasing its pathogenicity. This suggests that further improvement in oral health can positively impact glycemic control in diabetic patients. Obesity has also been found to be associated with the oral microbiome (Gasmi Benahmed et al., 2021), with alterations in salivary bacterial composition observed in overweight children (Balakrishnan et al., 2021). Bacterial species may serve as potential biomarkers for the development of overweight conditions. Oral bacteria may contribute to the pathogenesis of obesity. Although the association between oral microbial imbalance and the development of cardiovascular diseases is not yet robust, researchers have indeed demonstrated potential links between atherosclerosis and the oral microbiome (Tonelli et al., 2023). Specifically, several bacterial taxa in the oral and gut microbiota are associated with plasma cholesterol levels. The oral-gut micriobiota may be correlated with biomarkers of atherosclerotic disease (Isoshima et al., 2021). An increasing number of gastrointestinal (GI) diseases have been found to be associated with the oral microbiome (Park et al., 2021), with inflammatory bowel disease (IBD) being one of the earliest identified. Patients with IBD often exhibit various oral manifestations (Elmaghrawy et al., 2022), such as aphthous stomatitis and oral ulcers, suggesting a potential link between the oral microbiome and these presentations. The salivary microbiota colonize the gut and effectively induce chronic intestinal inflammation (Atarashi et al., 2017), particularly strains of the *Klebsiella*. Additionally, 54% of patients with liver cirrhosis harbor an abundance of taxonomically assigned gut bacterial species originating from the oral cavity (Qin et al., 2014), suggesting that the microbial source of liver cirrhosis is the translocation from the oral cavity to the gut. Individuals with periodontal disease, tooth loss, or oral bacterial infections have an increased risk of developing gastrointestinal cancers (He et al., 2015a). In a direct assessment of the oral metagenome-based microbiome in oral samples, the carriage of oral pathogens *Porphyromonas gingivalis (P. gingivalis)* and *Actinobacillus actinomycetemcomitans (A. actinomycetemcomitans)* is associated with an increased risk of pancreatic cancer (Herremans et al., 2022), while the *Leptotrichia* is associated with a reduced risk. These oral bacteria may serve as easily accessible, non-invasive biomarkers for subsequent pancreatic cancer risk, helping to identify high-risk populations. The oral microbiome is closely related to human immune system function and is therefore associated with autoimmune diseases such as rheumatoid arthritis (RA) (O'Shea et al., 2013), as well as multisystem immune diseases like human immunodeficiency virus (HIV) infection (Starr et al., 2018). Consequently, further investigation of the connections between the oral microbiome and systemic diseases is warranted, as it may unveil new opportunities for enhancing human health and well-being.

Third, our keyword analysis reveals the increasing prominence of research on the oral microbiome’s role in tumorigenesis (Hayes et al., 2018), particularly in the context of oral cancer (Liu et al., 2022). This emerging research area offers significant potential for identifying novel diagnostic and prognostic markers, as well as for developing targeted therapies and preventive measures. The carcinogenic role mediated by the oral microbiome has been found to satisfy or induce most hallmarks of cancer. In fact, several oral microorganisms (such as *P. gingivalis, Fusobacterium nucleatum (F. nucleatum), and Prevotella intermedia (P. intermedia))* associated with periodontal disease have been linked to an increased risk of developing gastrointestinal cancers (Park et al., 2021). Moreover, compared to healthy controls, the microbial samples of oral squamous cell carcinoma (OSCC) patients show elevated abundance of *P. gingivalis, F. nucleatum*, and *Alloprevotella* (Zhang et al., 2019). Mouse models have also demonstrated that *P. gingivalis* increases tumor heterogeneity and size, promoting tumor progression (Qi et al., 2020; Wen et al., 2020). Interestingly, *Streptococcus anginosus (S. anginosus)* is not only detected more frequently in esophageal cancer samples compared to oral cancer but also exhibits higher relative abundance (Morita et al., 2003). Furthermore, over half of the esophageal cancer patients carry a high abundance of *P. gingivalis*, indicating a strong association between the oral microbiome and the development of esophageal cancer (Zhao et al., 2020). Concurrently, the presence of *F. nucleatum* in tumors is associated with a poorer prognosis (Brennan and Garrett, 2019). Given the low early detection rates and high mortality rates characteristic of pancreatic cancer (Herremans et al., 2022), many research groups have been investigating the feasibility of using specific members of the oral microbiome as biomarkers. We also believe that ectopic colonization of oral microbiota in the gastrointestinal mucosa may be essential for dysregulation of the host microenvironment. Microbiota-mediated carcinogenic mechanisms include the generation of pro-inflammatory conditions, immune suppression, and inhibition of cell apoptosis (Pan et al., 2009; Feller et al., 2013; Atarashi et al., 2017; Rojas-Tapias et al., 2022). In this manner, oral microbiota can induce various cancer hallmarks. In addition to promoting cancer development and progression, the microbiome is also implicated in mediating resistance to anti-cancer treatments. As more information emerges regarding the relationships between the oral microbiome and various cancers, it may become possible to utilize its members as biomarkers for disease.

Moreover, the increasing focus on the oral microbiome’s impact on oral health, inflammation, and immune responses further emphasizes the need for a more comprehensive understanding of the microbiome’s role in oral and overall health. Many oral microbiota associated with carcinogenesis are pathogenic or conditionally pathogenic, which can induce chronic inflammatory responses (Li et al., 2022). An imbalance in the oral microbiome leads to a significant increase in the production of well-known inflammatory mediators and effector molecules (Costa et al., 2023). The local concentrations of cytokines such as interleukin-1β (IL-1β), interleukin-6 (IL-6), and matrix metalloproteinases (MMPs) are markedly elevated, serving as drivers of inflammation progression (Baeza et al., 2016). These cytokines typically activate inflammatory signaling pathways, such as nuclear factor-κB (NF-κB), Wnt, and JAK-STAT3 cascades, which are also genetically linked to carcinogenic effects (Johnson et al., 2018), potentially explaining the impact of the microbiome on cancer development to some extent. Similar to the gut microbiome, substances produced by the oral microbiota may also be associated with carcinogenic effects. Reactive oxygen species (ROS) are cellular metabolic byproducts generated during inflammation processes induced by the microbiome (Zhang et al., 2021). Their contributions to the pathogenesis of cancer have been demonstrated in various processes (Helmink et al., 2019), including cellular transformation, tumor survival, invasion, angiogenesis, and metastasis. Lipopolysaccharide (LPS) is a common pathogenic substance shared by many anaerobic oral bacteria (Dewhirst et al., 2010). Its ability to activate inflammatory processes is extensively linked to inflammation-associated carcinogenic mechanisms. Many cancer-related cytokines, such as IL-1β, IL-6, and TNF-α, are elevated during oral infections due to LPS stimulation (Rathinam et al., 2019). They may also be implicated in tumorigenesis, as their ability to activate inflammatory signaling and MMP9 could potentially promote tumor development and progression to some extent (Zhang et al., 2020). In addition to understanding the oral microbiome’s role in health and disease, researchers are also exploring its potential as a source of novel bioactive compounds, such as antimicrobials, anti-inflammatories, and anticancer agents.

Finally, the analysis of key genes and diseases associated with the oral microbiome also underscores the shift from traditional inflammatory pathways to more diverse fields such as immunity and cancer. This trend highlights the complex and multifaceted nature of the oral microbiome’s impact on human health, as well as the need for a more nuanced understanding of its molecular mechanisms and interactions.

In conclusion, the results of our bibliometric analysis indicate that the field of oral microbiome research is rapidly evolving and expanding, with a growing emphasis on the interplay between the oral microbiome and systemic diseases, immunity, and cancer. These emerging research areas offer significant potential for advancing our understanding of the oral microbiome’s role in human health and disease and may pave the way for novel therapeutic and preventive strategies. As the field continues to grow and develop, it is essential for researchers to remain cognizant of these trends and to build upon the existing body of knowledge in order to drive further progress and innovation in oral microbiome research.

Future research in the oral microbiome field should prioritize fostering collaboration between institutions and researchers, both nationally and internationally. Encouraging the sharing of resources, data, and expertise will be crucial to promoting the development of a more comprehensive understanding of the oral microbiome and its impact on human health. Furthermore, interdisciplinary approaches that combine cutting-edge technologies and methodologies from various fields, such as genomics, metagenomics, and metabolomics, should be emphasized. This will enable researchers to unravel the complex interactions between the oral microbiome and host, and subsequently develop targeted therapies and preventive strategies. Additionally, as our understanding of the oral microbiome’s role in systemic diseases and conditions continues to expand, researchers should focus on elucidating the underlying molecular mechanisms that drive these associations. This will not only deepen our understanding of the oral microbiome’s role in disease pathogenesis but also pave the way for the development of novel therapeutic targets and preventive measures.

In summary, the oral microbiome field is at the forefront of significant advancements and breakthroughs, with the potential to revolutionize our understanding of human health and disease. By maintaining a focus on collaboration, interdisciplinary approaches, and a comprehensive understanding of the oral microbiome’s role in systemic diseases and conditions, researchers in this field can contribute to the development of novel therapies and preventive strategies that will ultimately improve human oral health and overall well-being.

## 5 Conclusion

In conclusion, our bibliometric analysis reveals a rapidly evolving and expanding oral microbiome research landscape, emphasizing the interplay between oral microbiome, systemic diseases, immunity, and cancer. This multidisciplinary and integrative approach offers potential advancements in understanding the oral microbiome’s role in human health and disease, paving the way for innovative therapeutic and preventive strategies. Researchers must stay abreast of these trends and build on the existing knowledge to drive further progress in this field. Future oral microbiome research should prioritize fostering national and international collaborations, facilitating resource, data, and expertise sharing to promote a comprehensive understanding of the oral microbiome’s impact on human health. Emphasizing interdisciplinary approaches that integrate cutting-edge technologies and methodologies, such as genomics, metagenomics, and metabolomics, will help researchers unravel complex interactions between the oral microbiome and host, enabling the development of targeted therapies and preventive strategies. As the role of the oral microbiome in systemic diseases and conditions continues to expand, researchers should focus on elucidating underlying molecular mechanisms driving these associations, deepening our understanding of disease pathogenesis and enabling the development of novel therapeutic targets and preventive measures. In summary, the oral microbiome field holds significant potential to revolutionize our understanding of human health and disease. By emphasizing collaboration, interdisciplinary approaches, and a comprehensive understanding of the oral microbiome’s role in systemic diseases and conditions, researchers can contribute to the development of novel therapies and preventive strategies, ultimately improving human oral health and overall well-being.

## Data availability statement

The original contributions presented in the study are included in the article/**Supplementary Material**. Further inquiries can be directed to the corresponding author.

## Author contributions

ZL and RF wrote the manuscript. ZL, RF, XH and XW analyzed the data and drew pictures. ZL and LZ reviewed and revised the manuscript. All authors contributed to the article and approved the submitted version.

## References

[B1] Aas J. A. Paster B. J. Stokes L. N. Olsen I. Dewhirst F. E. (2005). Defining the normal bacterial flora of the oral cavity. J. Clin. Microbiol. 43 (11), 5721–5732. doi: 10.1128/JCM.43.11.5721-5732.2005 16272510 PMC1287824

[B2] Atarashi K. Suda W. Luo C. Kawaguchi T. Motoo I. Narushima S.. (2017). Ectopic colonization of oral bacteria in the intestine drives T (H)1 cell induction and inflammation. Science 358 (6361), 359–365. doi: 10.1126/science.aan4526 29051379 PMC5682622

[B3] Baeza M. Garrido M. Hernandez-Rios P. Dezerega A. Garcia-Sesnich J. Strauss F.. (2016). Diagnostic accuracy for apical and chronic periodontitis biomarkers in gingival crevicular fluid: an exploratory study. J. Clin. Periodontol 43 (1), 34–45. doi: 10.1111/jcpe.12479 26556177

[B4] Baker J. L. Bor B. Agnello M. Shi W. He X. (2017). Ecology of the oral microbiome: beyond bacteria. Trends Microbiol. 25 (5), 362–374. doi: 10.1016/j.tim.2016.12.012 28089325 PMC5687246

[B5] Baker J. L. Hendrickson E. L. Tang X. Lux R. He X. Edlund A.. (2019). Klebsiella and providencia emerge as lone survivors following long-term starvation of oral microbiota. Proc. Natl. Acad. Sci. U.S.A. 116 (17), 8499–8504. doi: 10.1073/pnas.1820594116 30975748 PMC6486781

[B6] Balachandran M. Cross K. L. Podar M. (2020). Single-cell genomics and the oral microbiome. J. Dent. Res. 99 (6), 613–620. doi: 10.1177/0022034520907380 32091935 PMC7243419

[B7] Balakrishnan B. Selvaraju V. Chen J. Ayine P. Yang L. Babu J. R.. (2021). Ethnic variability associating gut and oral microbiome with obesity in children. Gut Microbes 13 (1), 1–15. doi: 10.1080/19490976.2021.1882926 PMC7894456 33596768

[B8] Brennan C. A. Garrett W. S. (2019). Fusobacterium nucleatum - symbiont, opportunist and oncobacterium. Nat. Rev. Microbiol. 17 (3), 156–166. doi: 10.1038/s41579-018-0129-6 30546113 PMC6589823

[B9] Chipashvili O. Utter D. R. Bedree J. K. Ma Y. Schulte F. Mascarin G.. (2021). Episymbiotic saccharibacteria suppresses gingival inflammation and bone loss in mice through host bacterial modulation. Cell Host Microbe 29 (11), 1649–1662 e1647. doi: 10.1016/j.chom.2021.09.009 34637779 PMC8595704

[B10] Colombo A. P. V. Tanner A. C. R. (2019). The role of bacterial biofilms in dental caries and periodontal and peri-implant diseases: a historical perspective. J. Dent. Res. 98 (4), 373–385. doi: 10.1177/0022034519830686 30890060

[B11] Costa S. A. Ribeiro C. C. C. Leite F. R. M. Peres M. A. Souza S. F. C. Nascimento G. G. (2023). Chronic oral diseases burden: the confluence of caries and periodontitis throughout life. J. Clin. Periodontol 50 (4), 452–462. doi: 10.1111/jcpe.13761 36549902

[B12] Dewhirst F. E. Chen T. Izard J. Paster B. J. Tanner A. C. Yu W. H.. (2010). The human oral microbiome. J. Bacteriol 192 (19), 5002–5017. doi: 10.1128/JB.00542-10 20656903 PMC2944498

[B13] Elmaghrawy K. Fleming P. Fitzgerald K. Cooper S. Dominik A. Hussey S.. (2022). The oral microbiome in treatment naive paediatric IBD patients exhibits dysbiosis related to disease severity that resolves following therapy. J. Crohns Colitis. 17 (4), 553–564. doi: 10.1093/ecco-jcc/jjac155 PMC10115232 36239621

[B14] Farias da Cruz M. Barauna Magno M. Alves Jural L. Pimentel T. C. Masterson Tavares Pereira Ferreira D. Almeida Esmerino E.. (2022). Probiotics and dairy products in dentistry: a bibliometric and critical review of randomized clinical trials. Food Res. Int. 157, 111228. doi: 10.1016/j.foodres.2022.111228 35761544

[B15] Feller L. Altini M. Lemmer J. (2013). Inflammation in the context of oral cancer. Oral. Oncol. 49 (9), 887–892. doi: 10.1016/j.oraloncology.2013.07.003 23910564

[B16] Flemer B. Warren R. D. Barrett M. P. Cisek K. Das A. Jeffery I. B.. (2018). The oral microbiota in colorectal cancer is distinctive and predictive. Gut 67 (8), 1454–1463. doi: 10.1136/gutjnl-2017-314814 28988196 PMC6204958

[B17] Gaffen S. L. Moutsopoulos N. M. (2020). Regulation of host-microbe interactions at oral mucosal barriers by type 17 immunity. Sci. Immunol. 5 (43), eaau4594. doi: 10.1126/sciimmunol.aau4594 31901072 PMC7068849

[B18] Gao L. Xu T. Huang G. Jiang S. Gu Y. Chen F. (2018). Oral microbiomes: more and more importance in oral cavity and whole body. Protein Cell 9 (5), 488–500. doi: 10.1007/s13238-018-0548-1 29736705 PMC5960472

[B19] Gasmi Benahmed A. Gasmi A. Dosa A. Chirumbolo S. Mujawdiya P. K. Aaseth J.. (2021). Association between the gut and oral microbiome with obesity. Anaerobe 70, 102248. doi: 10.1016/j.anaerobe.2020.102248 32805390

[B20] Gebrayel P. Nicco C. Al Khodor S. Bilinski J. Caselli E. Comelli E. M.. (2022). Microbiota medicine: towards clinical revolution. J. Transl. Med. 20 (1), 111. doi: 10.1186/s12967-022-03296-9 35255932 PMC8900094

[B21] Graves D. T. Correa J. D. Silva T. A. (2019). The oral microbiota is modified by systemic diseases. J. Dent. Res. 98 (2), 148–156. doi: 10.1177/0022034518805739 30359170 PMC6761737

[B22] Hayes R. B. Ahn J. Fan X. Peters B. A. Ma Y. Yang L.. (2018). Association of oral microbiome with risk for incident head and neck squamous cell cancer. JAMA Oncol. 4 (3), 358–365. doi: 10.1001/jamaoncol.2017.4777 29327043 PMC5885828

[B23] He J. Li Y. Cao Y. Xue J. Zhou X. (2015a). The oral microbiome diversity and its relation to human diseases. Folia Microbiol. (Praha) 60 (1), 69–80. doi: 10.1007/s12223-014-0342-2 25147055

[B24] He X. McLean J. S. Edlund A. Yooseph S. Hall A. P. Liu S. Y.. (2015b). Cultivation of a human-associated TM7 phylotype reveals a reduced genome and epibiotic parasitic lifestyle. Proc. Natl. Acad. Sci. U.S.A. 112 (1), 244–249. doi: 10.1073/pnas.1419038112 25535390 PMC4291631

[B25] Helmink B. A. Khan M. A. W. Hermann A. Gopalakrishnan V. Wargo J. A. (2019). The microbiome, cancer, and cancer therapy. Nat. Med. 25 (3), 377–388. doi: 10.1038/s41591-019-0377-7 30842679

[B26] Herremans K. M. Riner A. N. Cameron M. E. McKinley K. L. Triplett E. W. Hughes S. J.. (2022). The oral microbiome, pancreatic cancer and human diversity in the age of precision medicine. Microbiome 10 (1), 93. doi: 10.1186/s40168-022-01262-7 35701831 PMC9199224

[B27] Hosomi K. Saito M. Park J. Murakami H. Shibata N. Ando M.. (2022). Oral administration of blautia wexlerae ameliorates obesity and type 2 diabetes *via* metabolic remodeling of the gut microbiota. Nat. Commun. 13 (1), 4477. doi: 10.1038/s41467-022-32015-7 35982037 PMC9388534

[B28] Isoshima D. Yamashiro K. Matsunaga K. Taniguchi M. Matsubara T. Tomida S.. (2021). Microbiome composition comparison in oral and atherosclerotic plaque from patients with and without periodontitis. Odontology 109 (1), 239–249. doi: 10.1007/s10266-020-00524-w 32430725

[B29] Johnson D. E. O'Keefe R. A. Grandis J. R. (2018). Targeting the IL-6/JAK/STAT3 signalling axis in cancer. Nat. Rev. Clin. Oncol. 15 (4), 234–248. doi: 10.1038/nrclinonc.2018.8 29405201 PMC5858971

[B30] Kleinstein S. E. Nelson K. E. Freire M. (2020). Inflammatory networks linking oral microbiome with systemic health and disease. J. Dent. Res. 99 (10), 1131–1139. doi: 10.1177/0022034520926126 32459164 PMC7443998

[B31] Kunath B. J. Hickl O. Queiros P. Martin-Gallausiaux C. Lebrun L. A. Halder R.. (2022). Alterations of oral microbiota and impact on the gut microbiome in type 1 diabetes mellitus revealed by integrated multi-omic analyses. Microbiome 10 (1), 243. doi: 10.1186/s40168-022-01435-4 36578059 PMC9795701

[B32] Li Z. Liu Y. Zhang L. (2022). Role of the microbiome in oral cancer occurrence, progression and therapy. Microb. Pathog. 169, 105638. doi: 10.1016/j.micpath.2022.105638 35718272

[B33] Liu Y. Li Z. Qi Y. Wen X. Zhang L. (2022). Metagenomic analysis reveals a changing microbiome associated with the depth of invasion of oral squamous cell carcinoma. Front. Microbiol. 13. doi: 10.3389/fmicb.2022.795777 PMC8863607 35222330

[B34] Long J. Cai Q. Steinwandel M. Hargreaves M. K. Bordenstein S. R. Blot W. J.. (2017). Association of oral microbiome with type 2 diabetes risk. J. Periodontal Res. 52 (3), 636–643. doi: 10.1111/jre.12432 28177125 PMC5403709

[B35] Morita E. Narikiyo M. Yano A. Nishimura E. Igaki H. Sasaki H.. (2003). Different frequencies of streptococcus anginosus infection in oral cancer and esophageal cancer. Cancer Sci. 94 (6), 492–496. doi: 10.1111/j.1349-7006.2003.tb01471.x 12824872 PMC11160302

[B36] O'Shea J. J. Laurence A. McInnes I. B. (2013). Back to the future: oral targeted therapy for RA and other autoimmune diseases. Nat. Rev. Rheumatol 9 (3), 173–182. doi: 10.1038/nrrheum.2013.7 23419429 PMC4169143

[B37] Pan Y. Teng D. Burke A. C. Haase E. M. Scannapieco F. A. (2009). Oral bacteria modulate invasion and induction of apoptosis in HEp-2 cells by pseudomonas aeruginosa. Microb. Pathog. 46 (2), 73–79. doi: 10.1016/j.micpath.2008.10.012 19041936

[B38] Park S. Y. Hwang B. O. Lim M. Ok S. H. Lee S. K. Chun K. S.. (2021). Oral-gut microbiome axis in gastrointestinal disease and cancer. Cancers (Basel) 13 (9), 2124. doi: 10.3390/cancers13092124 33924899 PMC8125773

[B39] Pasolli E. Asnicar F. Manara S. Zolfo M. Karcher N. Armanini F.. (2019). Extensive unexplored human microbiome diversity revealed by over 150,000 genomes from metagenomes spanning age, geography, and lifestyle. Cell 176 (3), 649–662 e620. doi: 10.1016/j.cell.2019.01.001 30661755 PMC6349461

[B40] Peng X. Cheng L. You Y. Tang C. Ren B. Li Y.. (2022). Oral microbiota in human systematic diseases. Int. J. Oral. Sci. 14 (1), 14. doi: 10.1038/s41368-022-00163-7 35236828 PMC8891310

[B41] Qi Y. J. Jiao Y. L. Chen P. Kong J. Y. Gu B. L. Liu K.. (2020). Porphyromonas gingivalis promotes progression of esophageal squamous cell cancer *via* TGFbeta-dependent Smad/YAP/TAZ signaling. PloS Biol. 18 (9), e3000825. doi: 10.1371/journal.pbio.3000825 32886690 PMC7498034

[B42] Qin H. Li G. Xu X. Zhang C. Zhong W. Xu S.. (2022). The role of oral microbiome in periodontitis under diabetes mellitus. J. Oral. Microbiol. 14 (1), 2078031. doi: 10.1080/20002297.2022.2078031 35694215 PMC9176325

[B43] Qin N. Yang F. Li A. Prifti E. Chen Y. Shao L.. (2014). Alterations of the human gut microbiome in liver cirrhosis. Nature 513 (7516), 59–64. doi: 10.1038/nature13568 25079328

[B44] Rathinam V. A. K. Zhao Y. Shao F. (2019). Innate immunity to intracellular LPS. Nat. Immunol. 20 (5), 527–533. doi: 10.1038/s41590-019-0368-3 30962589 PMC7668400

[B45] Ritchie M. K. Johnson L. C. Clodfelter J. E. Pemble C. Fulp B. E. Furdui C. M.. (2016). Crystal structure and substrate specificity of human thioesterase 2: INSIGHTS INTO THE MOLECULAR BASIS FOR THE MODULATION OF FATTY ACID SYNTHASE. J. Biol. Chem. 291 (7), 3520–3530. doi: 10.1074/jbc.M115.702597 26663084 PMC4751392

[B46] Rojas-Tapias D. F. Brown E. M. Temple E. R. Onyekaba M. A. Mohamed A. M. T. Duncan K.. (2022). Inflammation-associated nitrate facilitates ectopic colonization of oral bacterium veillonella parvula in the intestine. Nat. Microbiol. 7 (10), 1673–1685. doi: 10.1038/s41564-022-01224-7 36138166 PMC9728153

[B47] Sedghi L. DiMassa V. Harrington A. Lynch S. V. Kapila Y. L. (2021). The oral microbiome: role of key organisms and complex networks in oral health and disease. Periodontol 2000 87 (1), 107–131. doi: 10.1111/prd.12393 34463991 PMC8457218

[B48] Shen L. Wang S. Dai W. Zhang Z. (2019). Detecting the interdisciplinary nature and topic hotspots of robotics in surgery: social network analysis and bibliometric study. J. Med. Internet Res. 21 (3), e12625. doi: 10.2196/12625 30912752 PMC6454338

[B49] Starr J. R. Huang Y. Lee K. H. Murphy C. M. Moscicki A. B. Shiboski C. H.. (2018). Oral microbiota in youth with perinatally acquired HIV infection. Microbiome 6 (1), 100. doi: 10.1186/s40168-018-0484-6 29855347 PMC5984365

[B50] Sureda A. Daglia M. Arguelles Castilla S. Sanadgol N. Fazel Nabavi S. Khan H.. (2020). Oral microbiota and alzheimer's disease: do all roads lead to Rome? Pharmacol. Res. 151, 104582. doi: 10.1016/j.phrs.2019.104582 31794871

[B51] Takahashi N. (2015). Oral microbiome metabolism: from "Who are they?" to "What are they doing?". J. Dent. Res. 94 (12), 1628–1637. doi: 10.1177/0022034515606045 26377570

[B52] Tonelli A. Lumngwena E. N. Ntusi N. A. B. (2023). The oral microbiome in the pathophysiology of cardiovascular disease. Nat. Rev. Cardiol, 10.1038/s41569-022-00825-3. doi: 10.1038/s41569-022-00825-3 36624275

[B53] van Eck N. J. Waltman L. (2010). Software survey: VOSviewer, a computer program for bibliometric mapping. Scientometrics 84 (2), 523–538. doi: 10.1007/s11192-009-0146-3 20585380 PMC2883932

[B54] Wade W. G. (2013). The oral microbiome in health and disease. Pharmacol. Res. 69 (1), 137–143. doi: 10.1016/j.phrs.2012.11.006 23201354

[B55] Wen L. Mu W. Lu H. Wang X. Fang J. Jia Y.. (2020). Porphyromonas gingivalis promotes oral squamous cell carcinoma progression in an immune microenvironment. J. Dent. Res. 99 (6), 666–675. doi: 10.1177/0022034520909312 32298192

[B56] Xiao E. Mattos M. Vieira G. H. A. Chen S. Correa J. D. Wu Y.. (2017). Diabetes enhances IL-17 expression and alters the oral microbiome to increase its pathogenicity. Cell Host Microbe 22 (1), 120–128 e124. doi: 10.1016/j.chom.2017.06.014 28704648 PMC5701758

[B57] Xu X. Chen F. Huang Z. Ma L. Chen L. Pan Y.. (2018). Meeting report: a close look at oral biofilms and microbiomes. Int. J. Oral. Sci. 10 (3), 28. doi: 10.1038/s41368-018-0030-1 30111787 PMC6093876

[B58] Zhang H. Liu L. Jiang C. Pan K. Deng J. Wan C. (2020). MMP9 protects against LPS-induced inflammation in osteoblasts. Innate Immun. 26 (4), 259–269. doi: 10.1177/1753425919887236 31726909 PMC7251795

[B59] Zhang L. Liu Y. Zheng H. J. Zhang C. P. (2019). The oral microbiota may have influence on oral cancer. Front. Cell Infect. Microbiol. 9. doi: 10.3389/fcimb.2019.00476 PMC6974454 32010645

[B60] Zhang Y. Wang X. Li H. Ni C. Du Z. Yan F. (2018). Human oral microbiota and its modulation for oral health. BioMed. Pharmacother. 99, 883–893. doi: 10.1016/j.biopha.2018.01.146 29710488

[B61] Zhang B. Yang Y. Yi J. Zhao Z. Ye R. (2021). Hyperglycemia modulates M1/M2 macrophage polarization *via* reactive oxygen species overproduction in ligature-induced periodontitis. J. Periodontal Res. 56 (5), 991–1005. doi: 10.1111/jre.12912 34190354

[B62] Zhao Q. Yang T. Yan Y. Zhang Y. Li Z. Wang Y.. (2020). Alterations of oral microbiota in Chinese patients with esophageal cancer. Front. Cell Infect. Microbiol. 10. doi: 10.3389/fcimb.2020.541144 PMC7609410 33194789

[B63] Zheng X. Liu R. Zhou C. Yu H. Luo W. Zhu J.. (2021). ANGPTL4-mediated promotion of glycolysis facilitates the colonization of fusobacterium nucleatum in colorectal cancer. Cancer Res. 81 (24), 6157–6170. doi: 10.1158/0008-5472.CAN-21-2273 34645607 PMC9397643

[B64] Zhou X. Hao Y. Peng X. Li B. Han Q. Ren B.. (2021). The clinical potential of oral microbiota as a screening tool for oral squamous cell carcinomas. Front. Cell Infect. Microbiol. 11. doi: 10.3389/fcimb.2021.728933 PMC8416267 34485181

