# Supplementary material for: A decade of progress: bibliometric analysis of trends and hotspots in oral microbiome research (2013-2022)
Source: Front Cell Infect Microbiol. 2023 May 12;13:1195127. doi: 10.3389/fcimb.2023.1195127 (PMC10213461; doi:10.3389/fcimb.2023.1195127)
Supplement: Supplementary file 1 [file Table_1.docx]

Supplementary Material

A Decade of Progress: Bibliometric Analysis of Trends and Hotspots in Oral Microbiome Research (2013-2022)

Zhengrui Li^1,2,3,4,5,6,7^, Rao Fu^1,2,3,4,5,6,7^, Xufeng Huang^8^, Xutao Wen^1,2,3,4,5,6,7^, Ji'an Liu^1,2,3,4,5,6,7^, Ling Zhang^1,2,3,4,5,6,7,^*

^1^ Department of Oral and Maxillofacial-Head and Neck Oncology, Shanghai Ninth People’s Hospital, Shanghai Jiao Tong University School of Medicine, Shanghai, China;

^2^ College of Stomatology, Shanghai Jiao Tong University, Shanghai, China;

^3^ National Center for Stomatology, Shanghai, China;

^4^ National Clinical Research Center for Oral Diseases, Shanghai, China;

^5^ Shanghai Key Laboratory of Stomatology, Shanghai, China;

^6^ Shanghai Research Institute of Stomatology, Shanghai, China;

^7^ Shanghai Center of Head and Neck Oncology Clinical and Translational Science, Shanghai, China.

^8^ Faculty of Dentistry, University of Debrecen, Hungary.

*** Correspondence:** Prof. Ling Zhang: topgun1128@163.com

Table 1. 10 countries with the largest number of oral microbiome research results.

| Rank | Country | Count | Citations | Avg. citations |
| --- | --- | --- | --- | --- |
| 1 | USA | 1071(33.43%) | 35769 | 33.3978 |
| 2 | China | 636(19.85%) | 11835 | 18.6085 |
| 3 | United Kingdom | 238(7.43%) | 8915 | 37.458 |
| 4 | Italy | 199(6.21%) | 3963 | 19.9146 |
| 5 | Japan | 168(5.24%) | 2860 | 17.0238 |
| 6 | Brazil | 165(5.15%) | 2943 | 17.8364 |
| 7 | Germany | 158(4.93%) | 3838 | 24.2911 |
| 8 | Spain | 125(3.90%) | 2926 | 23.408 |
| 9 | Australia | 116(3.62%) | 3161 | 27.25 |
| 10 | Sweden | 108(3.37%) | 2880 | 26.6667 |

Table 2. 10 institutions with the largest number of oral microbiome research results.

| Rank | Institution | Country | Count | Citations | Avg. citations |
| --- | --- | --- | --- | --- | --- |
| 1 | Forsyth Institute | USA | 135 | 5598 | 41.4667 |
| 2 | [Sichuan University](https://www.so.com/link?m=bFWKLPv0swDenCYNX9zme/rG2wDPZx+U/hcZGQe41Btx3CHyBHOMdyngPaOv17IRnifMTO9qJo0xdCyNbx4JqJqtXGTx3uk0/Mlgkkc3ZK1np9bC18RryvxanKio=) | CN | 89 | 1546 | 17.3708 |
| 3 | [Harvard University](https://www.so.com/link?m=b0U62BXpBWI1RugHdl2RLyXCd0a8wcUfixH+5yHwHaAlV7rwjE3KSHGUM5sfNy1w+Q1DvWZkm/vHPVzU0U6Fl079lvecHXdRxH/gSpKW3nC3wx1SQ) | USA | 67 | 3503 | 52.2836 |
| 4 | [Peking University](https://www.so.com/link?m=baUfBHzaW5Aq5OE1HWdevaVVFbvDcyDB84sYdJ8HitGyEM3CdQdgPma3/DWCrVaBB9e5ukmaM6Sxhi4Vn3E1VwUsDkcdmaYGn3X++vCBVgSv+/Jrn271Bqw==) | CN | 60 | 969 | 16.15 |
| 5 | [University of Florida](https://www.so.com/link?m=bSyOI3X+ZZccfNccfSql0aOdQ9VCeQzXaHuWoFCpW0V74SWuQEDY/wMEZ6N8HqboPaGN4eCFgMSItrNmwR2yYNYWA81OXOf+/TY3EOPuqrO8s+/T8) | USA | 57 | 1605 | 28.1579 |
| 6 | University of Amsterdam | NL | 56 | 2288 | 40.8571 |
| 7 | University of Sao Paulo | BR | 56 | 858 | 15.3214 |
| 8 | New York University | USA | 51 | 2816 | 55.2157 |
| 9 | Harvard School of Dental Medicine | USA | 49 | 1596 | 32.5714 |
| 10 | Vrije University Amsterdam | NL | 49 | 2072 | 42.2857 |

Table 3. 10 authors with the largest number of oral microbiome research results.

| **Rank** | **Count** | **Author** | **Institution** | **Citations** | **Avg. Citations** | **H index** |
| --- | --- | --- | --- | --- | --- | --- |
| 1 | 32 | Paster, Bruce J. | The Forsyth Institute | 1093 | 34.1562 | 93 |
| 2 | 31 | Zhou, Xuedong | Sichuan University | 850 | 27.4194 | 36 |
| 3 | 24 | He, Xuesong | The Forsyth Institute | 871 | 36.2917 | 36 |
| 4 | 22 | Chen, Feng | Peking University | 755 | 34.3182 | 21 |
| 5 | 22 | Zaura, Egija | University of Amsterdam | 946 | 43 | 38 |
| 6 | 20 | Mclean, Jeffrey S. | University of Washington Seattle | 697 | 34.85 | 40 |
| 7 | 20 | Shi, Wenyuan | The Forsyth Institute | 1058 | 52.9 | 59 |
| 8 | 19 | Chen, Tsute | The Forsyth Institute | 825 | 43.4211 | 29 |
| 9 | 19 | Cheng, Lei | Sichuan University | 439 | 23.1053 | 50 |
| 10 | 18 | Brandt, Bernd W. | University of Amsterdam | 473 | 26.2778 | 27 |

Table 4. 10 highly-cited publications in the field of oral microbiome.

| Rank | Cite | Year | Title | Author | Journal | IF(2021) | JCR |
| --- | --- | --- | --- | --- | --- | --- | --- |
| 1 | 205 | 2018 | The oral microbiota: dynamic communities and host interactions. | Lamont RJ | Nature Reviews Microbiology | 78.297 | Q1 |
| 2 | 154 | 2019 | Reproducible, interactive, scalable and extensible microbiome data science using QIIME 2. | Bolyen E | Nature Biotechnology | 68.164 | Q1 |
| 3 | 142 | 2018 | Human oral microbiome and prospective risk for pancreatic cancer: a population-based nested case-control study. | Fan XZ | Gut | 31.793 | Q1 |
| 4 | 141 | 2016 | The oral microbiome - an update for oral healthcare professionals. | Kilian M | British Dental Journal | 2.727 | Q3 |
| 5 | 137 | 2013 | The oral microbiome in health and disease. | Wade WG | Pharmacological Research | 10.334 | Q1 |
| 6 | 119 | 2016 | DADA2: High-resolution sample inference from Illumina amplicon data. | Callahan BJ | Nature Methods | 47.990 | Q1 |
| 7 | 112 | 2016 | Biogeography of a human oral microbiome at the micron scale. | Welch JLM | PNAS | 12.779 | Q1 |
| 8 | 102 | 2018 | Oral microbiomes: more and more importance in oral cavity and whole body. | Gao L | Protein & Cell | 15.328 | Q1 |
| 9 | 101 | 2010 | The human oral microbiome. | Dewhirst FE | Journal of Bacteriology | 3.476 | Q3 |
| 10 | 97 | 2012 | Structure, function and diversity of the healthy human microbiome. | Huttenhower C | Nature | 69.504 | Q1 |
